# The effect of miRNA and autophagy on colorectal cancer

**DOI:** 10.1111/cpr.12900

**Published:** 2020-09-10

**Authors:** Jiali Long, Qinglian He, Yuting Yin, Xue Lei, Ziqi Li, Wei Zhu

**Affiliations:** ^1^ Department of Pathology Guangdong Medical University Dongguan China; ^2^ Department of Pathology the Eighth Affiliated Hospital Sun Yat‐Sen University Shenzhen China

**Keywords:** autophagy, colorectal cancer, microRNA, therapy

## Abstract

Colorectal cancer (CRC) has become a concern because of its high recurrence rate and metastasis rate, low early diagnosis rate and poor therapeutic effect. At present, various studies have shown that autophagy is closely connected with the occurrence and progression of CRC. Autophagy is a highly cytosolic catabolic process involved in lysosomes in biological evolution. Cells degrade proteins and damaged organelles by autophagy to achieve material circulation and maintain cell homeostasis. Moreover, microRNAs are key regulators of autophagy, and their mediated regulation of transcriptional and post‐transcriptional levels plays an important role in autophagy in CRC cells. This review focuses on the recent research advances of how autophagy and related microRNAs are involved in affecting occurrence and progression of CRC and provides a new perspective for the study of CRC treatment strategies.

## Graphical abstract

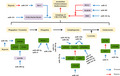

## 1 INTRODUCTION

Colorectal cancer (CRC) is the third leading cause of cancer‐related death worldwide.^1^ Old age, bad lifestyle and hereditary diseases are risk factors for CRC.^2^ Another risk factor is inflammatory bowel disease (IBD), including Crohn's disease (CD) and ulcerative colitis (UC).^3^ To date, treatments for CRC include some combination of surgery, radiation therapy, chemotherapy and targeted therapy.^4^ However, due to the inherent ability of CRC to become chemotherapy and radiation resistant, the combined‐modality therapy has failed to universally improve patients’ prognosis.^4^ In tumour therapy, apoptosis tolerance is an important mechanism of tumour resistance to treatment.^5^ Autophagy is able to prevent drugs‐induced apoptosis and promote tumour resistance.^6^ Nevertheless, autophagic cell death may also be a mode of death for apoptosis‐tolerant tumour cells.^7^ Therefore, autophagy has a dual effect on cancer progression and cancer treatment.

Autophagy is an evolutionarily conserved process that involves degradation of eukaryotic cellular components.^8^ Specifically, damaged or redundant proteins and dysfunctional cellular components are engulfed in the separation membrane and then extended into double‐membrane autophagosomes, followed by fusion of double‐membrane autophagosomes with lysosomes to form autophagosomes.^9^ It is subsequently degraded into simple ingredients to meet the energy and anabolic needs of the cells.^10^ Remarkably, the formation of autophagosome is regulated by autophagy‐related genes (ATGs), such as ATG12, ATG5 and microtubule‐associated protein light chain 3 (LC3).^11^ Autophagy is a stress response required for cellular survival.^12^ More extensive studies showed that autophagy mediates tumour survival by providing nutrients to stressed cancer cells.^13^ However, a report has demonstrated that activation of autophagy can result in cell death and inhibition of tumour progression.^14^ A growing body of evidence suggests that anti‐cancer therapies such as chemotherapy, radiation and targeted therapies can induce autophagy.^6^, ^15^ In addition, autophagy also plays a regulatory role in tumour cellular metabolic abnormalities and hypoxia.^16^, ^17^ Autophagy might have dual roles in tumour progression that may act as a suppressor at early stages and as a promoter at the advanced stages of CRC.^18^ Therefore, it is important to determine the regulative mechanisms of autophagy in CRC.

MicroRNAs (miRNAs) are non‐coding RNA molecules (20‐22 nucleotides in length) that primarily function to prevent mRNA translation or initiate mRNA degradation at the post‐transcriptional level via binding to the 3’ untranslated region (UTR) of their target mRNAs.^19^ Besides, some animal miRNAs may also target 5’ UTR and coding regions according to bioinformatics predictions and other experiments.^20^ Many investigations have shown that miRNAs are associated with numerous diseases such as tumours, autoimmune diseases, cardiac diseases and endocrine disorders.^21^ More studies have found that miRNAs are involved in tumour cell autophagy.^22^ Apart from the direct regulation between miRNAs and autophagy‐associated genes, accumulating evidences have indicated that autophagy is capable of regulating miRNA homeostasis via degrading the miRNA‐induced silencing complexes (miRISC).^23^ Studies have reported that miRNAs exert regulatory effect on the autophagy of CRC cells.^24^, ^25^ For example, miR‐216a regulates microtubule‐associated protein 1S (MAP1S)‐mediated autophagy inhibition and involves the transforming growth factor‐β (TGF‐β) pathway.^26^ Moreover, miR‐221 inhibits autophagy and targets tumour protein p53‐induced nuclear protein 1 (TP53INP1) in CRC cells.^27^ Overexpression of miR‐18a suppresses the activity of mammalian target of rapamycin complex 1 (mTORC1), thereby preventing the occurrence in CRC HCT116 cells by inducing autophagy.^28^ However, there is also evidence that miR‐338‐5p induces migration, invasion and metastasis of CRC by phosphatidylinositol 3‐kinase, catalytic subunit type3 (PIK3C3)‐related autophagy pathway.^29^ In addition, miRNAs are frequently dysregulated in chemoresistant cancers, shown to target autophagy‐related genes or modulators.^30^, ^31^ It is interesting to investigate the effect of miRNA and autophagy on CRC.

## 2 EFFECTS OF AUTOPHAGY AND MIRNA ON CHEMOTHERAPY OF CRC

In general, chemotherapeutic drugs are used as an adjunct to CRC.^32^ Chemical drugs repress cancer progression by preventing the cell cycle and inducing apoptosis.^32^ Oxaliplatin (OXA) is the third‐generation platinum compound and the first platinum compound to achieve a significant effect in the treatment of CRC.^33^ Nevertheless, despite the rapid reduction in tumour size after chemotherapy, cancer cells often develop resistance to OXA, leading to subsequent cancer recurrence and metastasis.^34^ Similarly, 5‐fluorouracil (5‐FU) is a widely used first‐line systemic chemotherapy drug. Its clinical therapeutic effect varies greatly among individuals, and drug resistance is considered to be the main reason for its failure to treat CRC.^35^ Some reports have indicated that the resistance of chemotherapeutics is highly correlated with the cytoprotective effects of autophagy.^36^, ^37^ In most cases, sustained drug exposure can induce an imbalance in the apoptotic pathway and lead to resistance to apoptosis.^38^ Additionally, chemotherapeutic drugs activate autophagy to protect cells from stress‐induced damage, thus promoting cancer cell resistance and reducing the efficiency of most anti‐cancer drugs.^39^, ^40^ Recently, some autophagy inhibitors have been shown to improve the efficacy of chemotherapeutic drugs for cancer treatment.^41^, ^42^ For example, inhibition of autophagy by 3‐methyladenine (3‐MA) and hydroxychloroquine (HCQ) can promote 5‐FU‐induced apoptosis in CRC cells.^43^ Therefore, enhancing apoptosis of cancer cells by inhibiting cytoprotective autophagy may be a promising strategy for adjuvant chemotherapy in CRC.

In vitro and in vivo experiments have illustrated that miR‐22 can inhibit autophagy and promote apoptosis to increase the sensitivity of 5‐FU treatment in CRC cells.^44^ B‐cell translocation gene 1 (BTG1), a new target of miR‐22, is a member of the anti‐proliferative gene family that regulates cell growth and differentiation and can reverse the inhibition of miR‐22‐induced autophagy.^45^ Therefore, miR‐22 may be considered as an important conversion factor between autophagy and apoptosis, and the sensitivity of 5‐FU may be regulated by post‐transcriptional silencing of BTG1.^44^ It has been reported that p53 is involved in apoptosis induced by 5‐FU and other chemotherapeutic agents, including DNA damage and induction of pro‐apoptotic genes such as Fas.^46^ Because p53‐mutant is less capable of inducing apoptosis, p53‐mutant CRC cells are more resistant to chemicals than p53‐wild‐type.^47^, ^48^ Compared with p53‐wide‐type CRC cells, accumulation of autophagosomes induced by 5‐FU treatment is more pronounced in p53‐mutant‐type CRC cells.^49^ The findings indicate that the mutant p53 regulates protective autophagy caused by chemotherapy or radiotherapy and has clinical implications.^50^, ^51^ Mammalian target of rapamycin (mTOR) significantly modulates the competition between autophagy and apoptosis, and its expression is regulated by miR‐338‐3p.^52^ It was showed that the miR‐338‐3p‐mTOR‐autophagy is regulated in a p53‐dependent manner and involved in the response to 5‐FU treatment.^31^ Chemokine (C‐X‐C motif) ligand 12 (CXCL12) and its receptor C‐X‐C chemokine receptor type 4 (CXCR4) play important roles in cancer growth, metastasis and invasion.^53^, ^54^ MiR‐125b is up‐regulated by activation of the CXCL12/CXCR4 axis, which in turn enhances CXCR4 expression.^30^ Study showed that miR‐125b confers 5‐FU resistance by increasing autophagy, displaying the increase of Beclin 1, microtubule‐associated protein light chain 3 II (LC3‐II) cleavage and autophagosome formation.^30^

It was observed that overexpression of miR‐409‐3p sensitizes CRC cells to OXA and restrains chemotherapy‐induced autophagy in a manner that depends on Beclin 1, suggesting that miR‐409‐3p is able to enhance the chemosensitivity of CRC cells by inhibiting Beclin 1‐mediated autophagy.^55^ Furthermore, expression of miR‐34a is down‐regulated in OXA‐resistant cells, whereas transfection of miR‐34a mimics enhances the efficacy of OXA by repressing autophagy and enhances the efficacy of OXA against OXA‐resistant CRC cells. At the same time, the autophagy inhibitor 3‐MA enhances the pro‐apoptotic effect of OXA‐resistant cells. These evidences have implicated that activation of autophagy protects CRC cells from OXA‐induced apoptosis by suppressing miR‐34a expression.^56^ Wu *et al* identified that miR‐27b‐3p inhibited the expression of ATG10 at the post‐transcriptional level, thus inhibiting autophagy to sensitize CRC cells to OXA in vivo and in vitro.^57^

In addition, emerging evidence demonstrated that chemotherapy against tumours require the involvement of the immune system.^58^ Once the tumour immunogenic cell death (ICD) is induced in chemotherapy, the prognosis is good.^59^ However, the formation of autophagy is thought to promote immune evasion.^59^ Damage‐associated molecular patterns (DAMPs), which are recognized by receptors on the surface of immune cells, are released by autophagic cell death, apoptotic and necrotic tumour cells. They can initiate an adaptive immune response either directly or indirectly.^60^ It determines to some extent whether cell death is ICD or tolerogenic cell death.^61^ Chemotherapy‐induced ICD is able to trigger DAMPs, such as the kinetics of choleretic surface exposure, the secretion of adenosine tri‐phosphate (ATP) and high mobility group box 1 (HMGB1).^62^ Evidence suggested that high expression of miR‐27a induced by chemotherapeutic drugs disrupts DAMP, silences apoptotic pathways, and enhances cell growth and survival potential. Moreover, the high expression of miR‐27a is involved in liver metastasis and worse prognosis.^63^ Similarly, CRC cells expressing low levels of miR‐27a undergoing drug‐induced ICD can stimulate efficient maturation of dendritic cells and secretion of cytokines, promoting immune activation and cell death.^63^ Notably, miR‐27a also reversely regulates autophagy, and apoptosis and autophagy are oriented in the same direction in cell models.^64^, ^65^ Consequently, it is of great significance to make a further study on the role of autophagy in the chemoresistance of CRC cells.

## 3 EFFECTS OF AUTOPHAGY AND MIRNA ON CHEMOTHERAPY OF CRC STEM CELLS

It is reported that miRNAs are capable of destroying the ability of autophagy to increase the chemosensitivity of CRC stem cells and inhibit the invasion.^65^, ^66^ Cancer stem cells (CSCs) are cancer cells that have characteristics associated with normal stem cells, and it has the ability to differentiate into all cell types in a particular cancer sample.^67^ Since the small subpopulation of CSCs persists in tumours, CSCs can cause tumour resistance, relapse and metastasis through self‐renewal and differentiation.^68^, ^69^ The autophagy mechanism of CRC stem cells has been identified as one of the major contributors to CRC resistance to chemotherapy and recurrence and metastasis.^39^ Mothers against decapentaplegic homolog 2 (Smad2), a downstream gene of the transforming growth factor beta (TGF‐β) signalling pathway, is associated with increased TGF‐β levels and poor prognosis, which leads to increased survival of metastatic cells and organ colonization in advanced CRC.^70^ In the experiment, hsa‐miR‐140‐5p directly inhibits the expression of Smad2 and regulates ATG12, and then it suppresses cell invasion, proliferation and induced cell cycle arrest. In addition, hsa‐miR‐140‐5p disrupts autophagy and inhibits the growth and metastasis of CRC stem cells in vivo and in vitro.^71^ Furthermore, it is showed that miR‐502 induces cell cycle arrest at both G1 and G2 checkpoints and is more prominent in wild‐type p53 HCT116 cells, and it can also restrain autophagy and reduce tumour growth by targeting Ras‐related protein RAB1B.^72^ These findings provide new insights into the effects of autophagy and miRNA on CRC chemotherapy.

## 4 EFFECTS OF AUTOPHAGY AND MIRNA ON RADIOTHERAPY OF CRC

Recent studies have suggested that deregulation of autophagy is related to radiation resistance of tumours, and miRNA expression patterns are involved in the modification of radiation therapy.^73^, ^74^ After irradiation (IR) therapy, the levels of miR‐214 in human CRC cells and peripheral blood are significantly decreased, while autophagy in CRC cells is induced.^25^ Further experiments showed that miR‐214 is able to inhibit ATG12‐induced autophagy and increase apoptosis, thus significantly increasing the radiosensitivity of CRC.^25^ These results indicate that miR‐214 achieves radioresistant effect by targeting autophagy‐related gene ATG12.^25^, ^75^ However, contrary experimental results have shown that increased abundance of miR‐183‐5p and decreased ATG5 levels are associated with poor prognosis of CRC, and miR‐183‐5p enhances radioresistance of CRC by directly targeting ATG5.^76^ Thus, further and deeper research is needed to clarify the role of miRNA and autophagy on radiotherapy in CRC.

Fibroblasts maintain the structural integrity of connective tissue by continuously secreting precursors of the extracellular matrix.^77^ Cancer‐associated fibroblast (CAF) secretes growth factors and interacts with tumour cells to provide nutrient support for tumour growth and enhance tumour metabolic regulation and immunity reaction.^78^ A study by Yang *et al* revealed that up‐regulation of miR‐31 can inhibit the expression of autophagy‐related genes Beclin 1, ATG, damage‐regulated autophagy modulator (DRAM) and LC3, and it can increase the radiosensitivity of CRC cells co‐cultured with CAF.^79^

## 5 EFFECTS OF AUTOPHAGY AND MIRNA ON METABOLISM AND HYPOXIA OF CRC

Compared with normal cells, cancer cells exhibit a metabolic phenotype characterized by increased glycolysis and significantly alter nutrient utilization, regardless of oxygen availability. The phenomenon termed the Warburg effect.^80^ Since the survival of cancer cells mainly depends on the rate of high glucose consumption and the elevation of glycolysis, the Warburg effect and glucose metabolism are important strategies for cancer treatment.^81^ In mammals, glucose metabolism can be regulated by controlling the expression of pyruvate kinase isozymes M1/M2 (PKM1/M2).^82^ Abnormal expression of PKM1/M2 is essential for maintaining the growth of cancer cells.^83^ The ectopic expression of miR‐124 induces apoptosis and autophagy in CRC.^84^ The knock‐down of polypyrimidine tract‐binding protein 1 (PTB1, also known as heterogeneous nuclear ribonucleoprotein I) is able to induce drastically apoptotic cell death which indicates PTB1 acts as an oncogene. In vitro experimental studies confirmed that miR‐124 targets PTB1 and regulates the ratio of PKM1/PKM2 to inhibit CRC growth.^84^ Furthermore, miR‐18a induces apoptosis of CRC cells by directly binding to the oncogene heterogeneous nuclear ribonucleoprotein A1 (hnRNP A1) via autophago‐lysosomal pathway.^85^, ^86^

Due to the rapid proliferation of tumour cells, hypoxia within the tumour is one of the most important features of solid tumours. Hypoxia is a well‐known inducer of autophagy which leads to cancer cells resistance to chemotherapy and radiotherapy.^87^, ^88^ Hypoxia‐inducible factor 1‐alpha (HIF‐1α) is an important factor regulating cell responses to hypoxia.^89^ Hypoxia‐induced autophagy is also involved in HIF‐1α‐mediated cell survival mechanisms.^90^ The study found that miR‐210 is continuously up‐regulated in CRC and promotes CRC migration and invasion. ^90^, ^91^ Hypoxia induces HIF‐1α and its downstream target miR‐210, which is able to restrain the expression of B‐cell lymphoma 2 (Bcl‐2) and enhance autophagy, thereby contributing to the radioresistance of CRC cells.^92^ Bcl‐2 exerts a dual function as an anti‐apoptotic, anti‐autophagic protein, and may be related to reactive oxygen species (ROS) levels.^93^ Similarly, under conditions of nutrient starvation, low levels of Bcl‐2 phosphorylation initially occur and survival are promoted by activation of autophagy, while higher levels of Bcl‐2 phosphorylation accelerate apoptosis as prolonged starvation time.^94^ Besides, a previous study has reported that miR‐20a is significantly down‐regulated under hypoxia in CRC cells, and overexpression of miR‐20a directly targets ATG5 and FIP200 (focal adhesion kinase family kinase‐interacting protein of 200 KDa) and alleviates hypoxia‐induced autophagy.^95^ Consequently, a better understanding the mechanisms of miRNA and autophagy in metabolism and hypoxia may be of potential value in improving the effectiveness of CRC treatment.

## 6 EFFECTS OF AUTOPHAGY AND MIRNA ON INFLAMMATORY BOWEL DISEASE

IBD is a group of inflammatory disorders of the colon and small intestine, of which CD and UC are the major types of inflammatory bowel disease.^96^ Due to the presence of symptoms such as abdominal pain, vomiting, diarrhoea, rectal bleeding and anaemia in IBD, it seriously affects the life quality of patients.^97^, ^98^ CRC is a recognized and worrying complication for patients with long‐term colonic inflammation.^3^ Unfortunately, the current global incidence of IBD is on the rise.^99^ The onset of IBD is primarily due to an abnormal immune response against luminal antigens and microbiota.^100^

**Figure 1:**
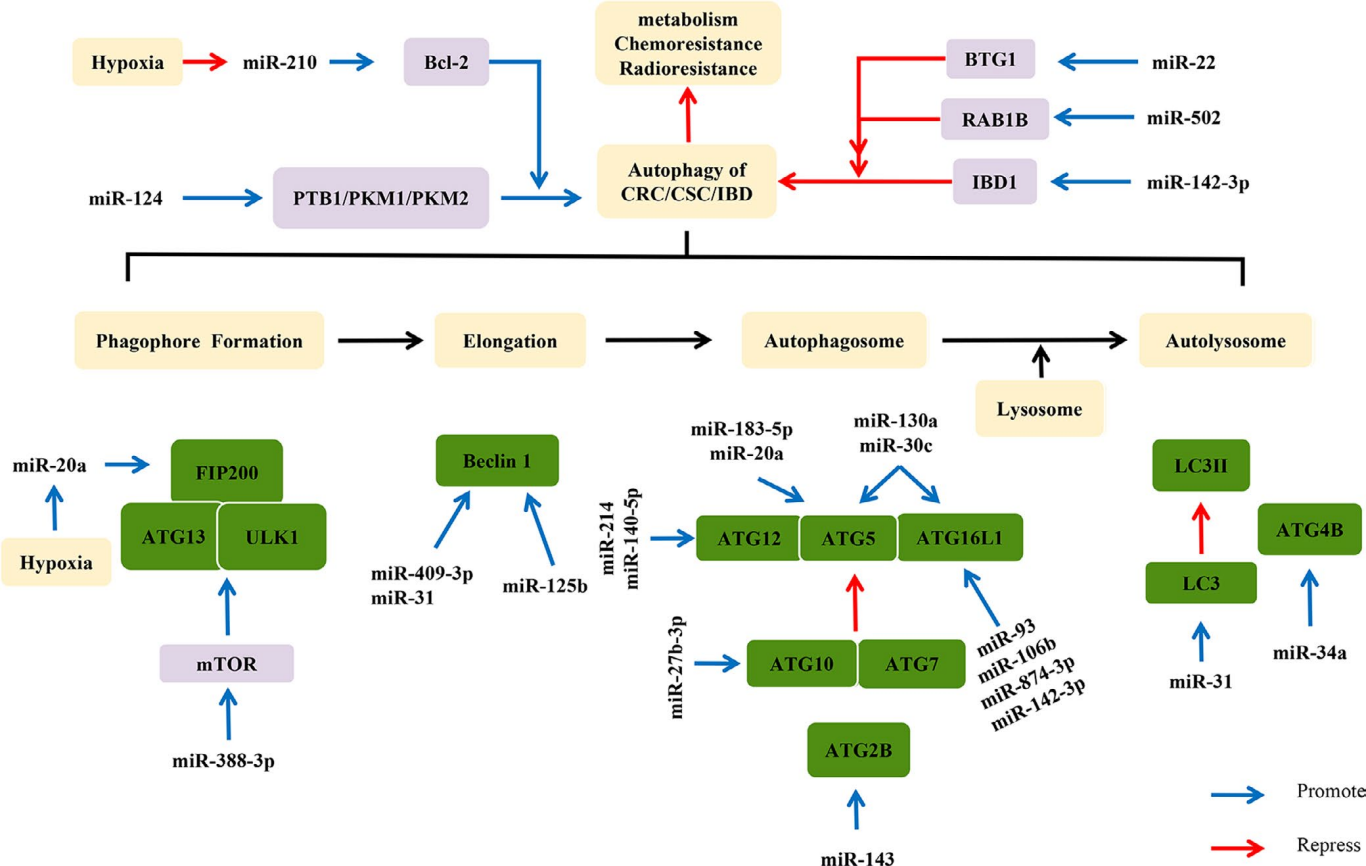
Regulatory relationship between miRNAs and autophagy in CRC. Autophagy consists of a series of activities, such as phagophore formation, elongation, autophagosome and fusion with lysosome to form autolysosome. MiRNAs exert dual regulatory effects on autophagy pathways related to CRC through indirect or direct pathways. In the figure, the purple box represents autophagy‐related proteins, which indirectly regulate the autophagy process (eg, Bcl‐2, mTOR), the green box represents autophagy‐associated proteins, which are directly involved in the occurrence or formation of autophagy (eg, Beclin 1, LC3), and the yellow box represents the cellular process

Dysfunctional autophagy is thought to be a contributing factor to many chronic inflammatory diseases including CD.^101^ Studies have pointed out that miRNA is capable of regulating autophagy‐related genes involved in the pathogenesis of IBD, such as ATG5, autophagy‐related gene 16‐like 1 (ATG16L1), autophagy‐related 2 homolog B (ATG2B) and immunity‐related GTPase family M protein (IRGM), while autophagy regulates miRNA homeostasis by degrading miRISC.^102^, ^103^ MiR‐142‐3p targets ATG16L1 and reduces the autophagic activity resulting from starvation‐induced cell death and apoptosis in CRC cells.^104^ At the same time, miR‐142‐3p can inhibit inflammatory bowel disease protein 1 (IBD1)‐dependent autophagy and effectively down‐regulate interleukin 8 (IL‐8) mRNA expression, further suggesting miR‐142‐3p exerts autophagy‐related effects in intestinal inflammation and CD.^104^ Highly expressed miR‐93 and miR‐106b can also target ATG16L1 in active CD to reduce autophagosome formation.^105^ Aylia *et al* extracted the total RNA from peripheral blood mononuclear cells of CD and UC patients and found miR‐874‐3p is the most differentially expressed. They further confirmed that miR‐874‐3p dysregulates autophagy by targeting ATG16L1.^106^ Above evidences have implicated unique panels of miRNAs in blood and tissue distinguishing CD and UC in varying regimes of disease activity. Additionally, both miR‐130a and miR‐30c are dysregulated in CD and considered to be key regulators of the autophagy pathway in innate immunity.^107^, ^108^ Nuclear factor kappa‐light‐chain‐enhancer of activated B cells (NF‐κB) protein is involved in the control of immune and inflammatory responses, developmental processes, cellular growth and apoptosis.^109^ It was demonstrated that enhanced autophagosome activity is effectively able to attenuate NF‐κB‐mediated inflammation.^110^ Experiments indicated that there is abnormal activation of NF‐κB pathway in miR‐143 overexpressed or ATG2B‐depleted CRC cell lines, suggesting that miR‐143 may suppress autophagy and increase inflammation reaction of the NF‐κB pathway in CD by targeting ATG2B.^111^ Therefore, further researches should be conducted to promote the clinical application of miRNAs in IBD. (Figure 1) (Table 1).

**Table 1:** MiRNAs regulating autophagy under different CRC conditions

Chemotherapy of CRC		
miRNA	Potential target of miRNA	Effect of miRNA on autophagy
smiR‐22	BTG1	Inhibition
miR‐338‐3p	mTOR	Inhibition
miR‐125b	CXCL12/CXCR4	Promotion
miR‐409‐3p	Beclin 1	Inhibition
miR‐34a	ATG4B	Inhibition
miR‐27b‐3p	ATG10	Inhibition
miR‐27a	PINK1	Inhibition
Chemotherapy of CRC stem cells		
miR‐140‐5p	ATG12	Inhibition
miR‐502	RAB1B	Inhibition
Radiotherapy of CRC		
miR‐214	ATG12	Inhibition
miR‐183‐5p	ATG5	Inhibition
miR‐31	Beclin 1, ATG, LC3	Inhibition
Metabolism and Hypoxia of CRC		
miR‐124	PTB1	Promotion
miR‐18a	hnRNP A1	Promotion
miR‐210	Bcl‐2	Promotion
miR‐20a	ATG5 and FIP200	Inhibition
Inflammatory bowel disease		
miR‐142‐3p	ATG16L1	Inhibition
miR‐142‐3p	IBD1	Inhibition
miR‐93	ATG16L1	Inhibition
miR‐106b	ATG16L1	Inhibition
miR‐874‐3p	ATG16L1	Inhibition
miR‐130a miR‐30c	ATG5 ATG16L1	Inhibition
miR‐143	ATG2B	Inhibition

Abbreviation: PINK1, PTEN‐induced putative kinase 1.

## 7 DISCUSSION AND CONCLUSION

Autophagy may perform a dual function in the progression of CRC.^18^ As described above, the autophagy inhibitors 3‐MA and HCQ are able to inhibit autophagy, promote 5‐FU‐induced CRC cell apoptosis and increase patient sensitivity to chemotherapy.^43^ However, some findings have revealed that the autophagy inducers rapamycin can reduce the migration capacity of CRC cells.^112^ Many miRNAs regulate autophagy under CRC stress conditions, including chemotherapy, radiation therapy, nutrient and hypoxia.^113^

MiRNAs are thought to increase/decrease the chemosensitization or radiosensitization by regulating the level of autophagy in CRC cells, which has potential value for the treatment of CRC. For example, miR‐22 restrains autophagy and promotes apoptosis, thereby improving the sensitivity of 5‐FU treatment in CRC cells.^44^ In contrast, miR‐183‐5p directly targets ATG5 to enhance the radioresistance of CRC.^76^

Noticeably, recent studies have reported that several miRNAs have been shown to target autophagy‐associated proteins in other tumours, regulating the occurrence and development of tumours. For instance, miR‐543 is a cancerous suppressor in ovarian cancer, inhibiting the expression of twist family bHLH transcription factor 1 (TWIST1).^114^ TWIST1 is not only a vital protein involved in tumour metastasis and invasion, but also its stability is modulated by p62, the substrate of autophagy.^115^ However, miR‐543 is a cancer‐promoting factor in CRC, which may be involved in regulating autophagy through the mTOR pathway to affect the effect of chemotherapy.^116^, ^117^, ^118^ MiRNA‐23b also has an inhibitory effect on non‐small cell carcinoma by inhibiting RUNX family transcription factor 2 (Runx2) ,^119^ while Runx2 has been shown to promote autophagy by increasing the acetylation of microtubule α‐tubulin subunits in advanced breast cancer cells.^120^ MiRNA‐23b in CRC promoted cell migration by down‐regulating forkhead box P2 (FOXP2).^121^ MiRNA‐142‐3p suppresses cellular proliferation and migration via directly acting on Rac family small GTPase 1 (Rac1) in bladder cancer.^122^ It has been reported that Rac1 can regulate autophagy.^123^ MiRNA‐142‐3p was found to function as a cancer‐promoting factor through Rac1 in CRC. By bioinformatics prediction, Rac1 has a miR‐142‐3p binding site in its 3'‐UTR.^124^ However, there is a positive correlation between Rac1 and miRNA‐142‐3p in CRC, and the authors believe that miRNA‐142‐3p may function through activating Rac1 indirectly.^124^ As can be seen from the above, although it has been found that miR‐543, miRNA‐23b and miRNA‐142‐3p are aberrantly expressed in CRC, the mechanism is not completely clear. There is a great possibility that it is related to autophagy, and more research is needed to explore this mechanism.

Since miRNA can be used as an influencing factor of autophagy, it may be a feasible research direction to study the upstream regulatory pathways of miRNA such as circular RNA (circRNA) and long non‐coding RNA (lncRNA). In the previous study, circHIPK3 which promoted OXA resistance in CRC through autophagy by sponging miR‐637 via miR‐637/STAT3/Bcl‐2/Beclin1 axis is up‐regulated in tissues from chemoresistant and recurrent CRC patients and correlated with tumour size, regional lymph node metastasis, distant metastasis and survival.^125^ Furthermore, lncRNAs have been reported to regulate chemoresistance.^126^ LncRNA small nucleolar RNA host gene 6 (SNHG6) is able to promote 5‐FU resistance through unc‐51 like autophagy activating kinase 1 (ULK1)‐induced autophagy by sponging miR‐26a‐5p in CRC cells.^127^ It has been proved that lncRNA metastasis‐associated lung adenocarcinoma transcript 1 (MALAT1), which promotes CRC chemotherapy resistance,^128^ can affect the expression of enhancer of zeste homolog 2 (EZH2) by up‐regulating miR‐363‐3p,^129^ and EZH2 is an essential regulatory factor that phosphorylates histone H2B and then increases autophagy.^130^ LncRNA H19 might work as a competing endogenous RNA (ceRNA) to sponge miR‐194‐5p and conferred 5‐FU resistance in CRC by promoting sirtuin1 (SIRT1)‐mediated autophagy.^131^ Moreover, lncRNA KCNQ1 opposite strand/antisense transcript 1 (KCNQ1OT1) enhances the chemoresistance of OXA in CRC by targeting the miR‐34a/ATG4B pathway.^56^ The therapeutic strategies of CRC besides exploring how miRNA and autophagy regulate the chemical sensitivity of CRC to drugs, the exploration of synthetic compounds such as miRNA mimic/inhibitor and natural component such as the anti‐tumour agent inositol hexaphosphate (IP6) which down‐regulated miR‐155 to modulate the autophagy‐related protein like HIF‐1α may also be a new approach.^132^

It is important to probe into the molecular mechanism of CRC treatment, but the method of detecting the effect of treatment cannot be ignored. In recent years, liquid biopsy, such as detection of circulating miRNAs in plasma and serum, has become a research hotspot, and detection of miRNAs in serum exosomes is also expected to become a predictive marker of chemoresistance in advanced CRC.^133^ However, it may also encounter problems of high cost or poor repeatability. Absolutely, future fundamental and clinical researches are required considering these limitations.

Additionally, IBD is a chronic complex disorder caused by a variety of factors.^134^ As mentioned above, autophagy‐related genes are regulated by multiple miRNAs in IBD and play a role in regulating inflammation, which is a complex network.^104^, ^105^ Therefore, miRNA can be regarded as a new diagnostic marker and therapeutic target. These evidences reveal underlying mechanisms of the pathophysiology and provide new diagnostic and therapeutic targets in IBD.

MiRNAs play an important role in the occurrence and development of CRC by regulating the level of autophagy in CRC cells. The exact mechanisms by which miRNAs‐regulated autophagy controls cancer occurrence and development have not been established. It appears to be dependent on the tumour microenvironment with a dual role of tumour promotion and inhibition. In fact, in addition to miRNA regulating autophagy, there are a few reports suggesting that autophagy may affect miRNA homeostasis.^135^ Autophagy has been found to degrade the enzymes Dicer and AGO2 during miRNA processing and maturation in several tumours.^23^, ^102^, ^136^, ^137^, ^138^ However, this phenomenon has not been reported in CRC. Therefore, it is necessary to determine how the autophagy mechanism exerts a dual effect in the CRC. Further research is needed to better understand the relationship between miRNA and autophagy in CRC and to produce potentially beneficial drugs for the prognosis and treatment of CRC.

## CONFLICT OF INTEREST

None.

## AUTHORS' CONTRIBUTIONS

JLL and QLH drafted the manuscript. YTY, XL and ZQL were involved in data gathering. WZ revised the manuscript. All authors read and approved the final manuscript.

## Data Availability

Data sharing is not applicable to this article as no new data were created or analysed in this study.
